# A demonstration of high field-of-view stability in hands-free echocardiography

**DOI:** 10.1186/s12947-020-00201-6

**Published:** 2020-05-29

**Authors:** Marloes Sjoerdsma, Louis S Fixsen, Thijs Schoots, Frans N van de Vosse, Richard GP Lopata

**Affiliations:** 1 Department of Biomedical Engineering, Eindhoven University of Technology, Groene Loper, Building 15, Eindhoven, The Netherlands; 2 Máxima Medical Centre, Veldhoven, The Netherlands

**Keywords:** Hands-free, Echocardiography, Wavelets, A-lines, Field-of-view, Stability, Probe fixation, Exercise, Strain, Complex wavelet structural similarity

## Abstract

**Background:**

Exercise stress echocardiography is clinically used to assess cardiovascular diseases. For accurate cardiac evaluation, a stable field-of-view is required. However, transducer orientation and position are difficult to preserve. Hands-free acquisitions might provide more consistent and reproducible results. In this study, the field-of-view stability and variability of hands-free acquisitions are objectively quantified in a comparison with manually obtained images, based on image structural and feature similarities. In addition, the feasibility and consistency of hands-free strain imaging is assessed.

**Methods:**

In twelve healthy males, apical and parasternal images were acquired hands-free, using a fixation device, and manually, during semi-supine exercise sessions. In the final ten seconds of every exercise period, the image structural similarity and cardiac feature consistency were computed using a steerable pyramid employing complex, oriented wavelets. An algorithm discarding images displaying lung artifacts was created. Hands-free strain consistency was analyzed.

**Results:**

Hands-free acquisitions were possible in 9 of the 12 subjects, whereas manually 10 out of 12 could be imaged. The image structural similarity was significantly improved in the hands-free apical window acquisitions (0.91 versus 0.82), and at least equally good in the parasternal window (0.90 versus 0.82). The change in curvature and orientation of the interventricular septum also appeared to be lower in the hands-free acquisitions. The variability in field-of-view was similar in both acquisitions. Longitudinal, septal strain was shown to be at least as consistent when obtained hands-free compared to manual acquisitions.

**Conclusions:**

The field-of-view was shown to be more or equally stable and consistent in the hands-free data in comparison to manually obtained images. The variability was similar, thus respiration- and exercise-induced motions were comparable for manual and hands-free acquisitions. Additionally, the feasibility of hands-free strain has been demonstrated. Furthermore, the results suggest the hands-free measurements to be more reproducible, though further analysis is required.

## Background

Stress echocardiography is employed in the clinic as a risk classifier on cardiovascular events in patients with suspected cardiac impairment. This diagnostic method provides insight in diastolic and systolic dysfunction during daily life activities, whereas unstressed measurements might underestimate the severity of anomalies. These cardiac impairments might include structural or functional abnormalities (e.g. hypertrophic cardiomyopathy, coronary artery disease, valve stenosis or leakage) leading to a reduction in cardiac output and/or an augmentation of intra-cardiac pressures. Furthermore, determination of lung water using B-lines in lung ultrasound [1, 2], left ventricular contractile reserve based on elastance [3], and Doppler-based assessment of coronary micro-circulation [4–6] are also included in the newer, more thoroughly implemented stress echocardiography protocols [7].

Exercise stress echocardiography is preferred over pharmacological stress, since exercise capacity is an important risk indicator and the target heart rate is less commonly achieved with pharmacological stress, which compromises its sensitivity [8, 9]. These tests are performed in an upright or supine position with an increasing exercise intensity until the target heart rate (85% of age-predicted maximum) is reached [10]. Usually in upright exercise, a higher maximum heart rate and workload are reached compared to exercise in a supine position [10–12]. In addition, upright exercise better resembles daily activities, is a better reflection of real-life exercise hemodynamics, and preload is decreased to a greater extend.

Continuous cardiac evaluation during exercise is strenuous and complicated, since the transducer has to be held in place by an observer. This is even more difficult during upright exercise. As a consequence, work-related musculoskeletal disorders are highly prevalent in echocardiography, due to a lack of variation in exams, small and precise repetitive movements, pressure application, and extended periods of poor static postures [13–15]. In addition, extensive imaging training is required, since the orientation and position of the transducer are difficult to reproduce or preserve manually. Hence, variance in the imaging plane is common, especially during exercise. The alternative option of imaging post (upright) exercise has a lower sensitivity, higher probability of disease underestimation, and results in large preload variations. Accordingly, although upright exercise provides physiological superior heart evaluation, supine exercise is still clinically preferred in case of functional measurements due to these constraints [10, 16].

In the clinic, regional contractile function is generally assessed by eyeballing of B-mode ultrasound images, whereas global measurements are usually performed using manual caliper measurements [17, 18]. Systolic function assessment is based on thickening of the wall, wall velocity, or ejection fraction in interval measurements [8, 10]. In diastolic evaluation, the filling volumes are monitored. Tissue Doppler velocity imaging is also a widely used method to determine cardiac wall velocity. Of all functional assessment methods, septal longitudinal strain has been shown to be very valuable, since it is able to differentiate between passive and active strain [19].

All assessment methods are adversely affected by out-of-plane motion, which is caused by upper body motion, cardiac contraction, respiration, and transducer motion. Hence, an identical and stable field of view (FOV) in the acquisitions is essential for early and correct diagnosis [20].

Ultrasound transducer fixation could resolve these exercise stress echocardiography issues. In existing hands-free echocardiography studies, novel probes were developed that were fixated into an external housing with screws, which employed rubber belts or adhesive patches for skin attachment [21, 22]. However, for clinical applications, an easily and quickly applicable fixation apparatus is required. This device must be firmly secured and patient friendly, whilst being suitable for a wide variety of ultrasound transducers.

Recently, a fixation device has been introduced in the clinic that satisfies these demands. The feasibility of this fixation device has been demonstrated for continuous cardiac acquisitions during ergometer stress tests in supine and upright positions in patients [23], and in intensive care where cardiac output was measured before and after a passive leg raising test [24]. Furthermore, the application of the fixation device in routine echocardiography results in a reduction of the total shoulder abduction time and the amount of forearm muscle contractions, without affecting the acquisition time being 5.1 versus 5.2 minutes in hands-free and manual acquisitions, respectively [25]. On average, the fixation of the probe in the desired window takes approximately 2.7 ±1.3 minutes [25]. Once positioned, rotation and angulation of the probe can be performed freely to obtain all the desired imaging planes (e.g. apical 2-, 3-, and 4-chamber view in the apical view, or the long- and short axis in the parasternal view). If a 3D probe is used, all imaging planes can be obtained without probe readjustment. For extra acquisitions in other acoustic windows, the probe can be de- and reattached to the fixation device in about a second.

An objective quantification of FOV stability is still lacking, and it is unknown whether probe fixation will yield a FOV that is stable enough for accurate and reproducible functional measurements. Therefore, the impact of probe fixation on the FOV stability is still unexplored, despite the importance of stability in the accurate clinical evaluation of cardiac diastolic and systolic function.

The aim of this paper is to give an objectively quantified demonstration of the FOV stability of hands-free acquisitions, which are acquired with a fixated transducer, and compare the FOV stability and variability to acquisitions obtained manually by a clinically trained cardiac sonographer. The acquisitions consist of apical and parasternal images obtained continuously in twelve healthy volunteers during semi-supine cycling exercise consisting of three successive exercise periods.

For an objective evaluation, algorithms were implemented to overcome inter-observer variability and bias. First, an algorithm was developed for automatic detection and elimination of frames showing lung-related artefacts, since only frames showing cardiac tissue were included in the FOV stability assessment. The FOV stability was quantified by analyzing the end-diastolic frames of the final ten seconds of each exercise period based on (i) their structural similarity, and (ii) cardiac feature consistency (i.e. interventricular septal curvature and orientation). Subsequently, the variability within the continuous measurements was compared between hands-free and manually acquired data to determine whether the clinically trained sonographer is able to correct for respiration-, and exercise-induced motions, whereas the fixation device will remain in the same position on the chest. Furthermore, consistency in hands-free, longitudinal, interventricular septal strain was compared to the strain curves from the manually obtained images.

## Methods

### Participants

Twelve healthy males participated in this study aged between 21 to 55 years old. Only males were included in this relatively small volunteer group, since their thorax shapes are roughly uniform. The subjects were healthy, had no musculoskeletal injuries and never experienced any cardiac disease related symptoms in rest or during exercise. Prior to participation, the subjects were fully informed on the research procedure and each gave written consent for the usage of their data for scientific purposes. The exercise protocol was assessed by the local Medical Ethics Committee of the Máxima Medical Centre, Veldhoven, The Netherlands, and ethical approval was waived.

### Data acquisition and exercise protocol

**Fig. 1:**
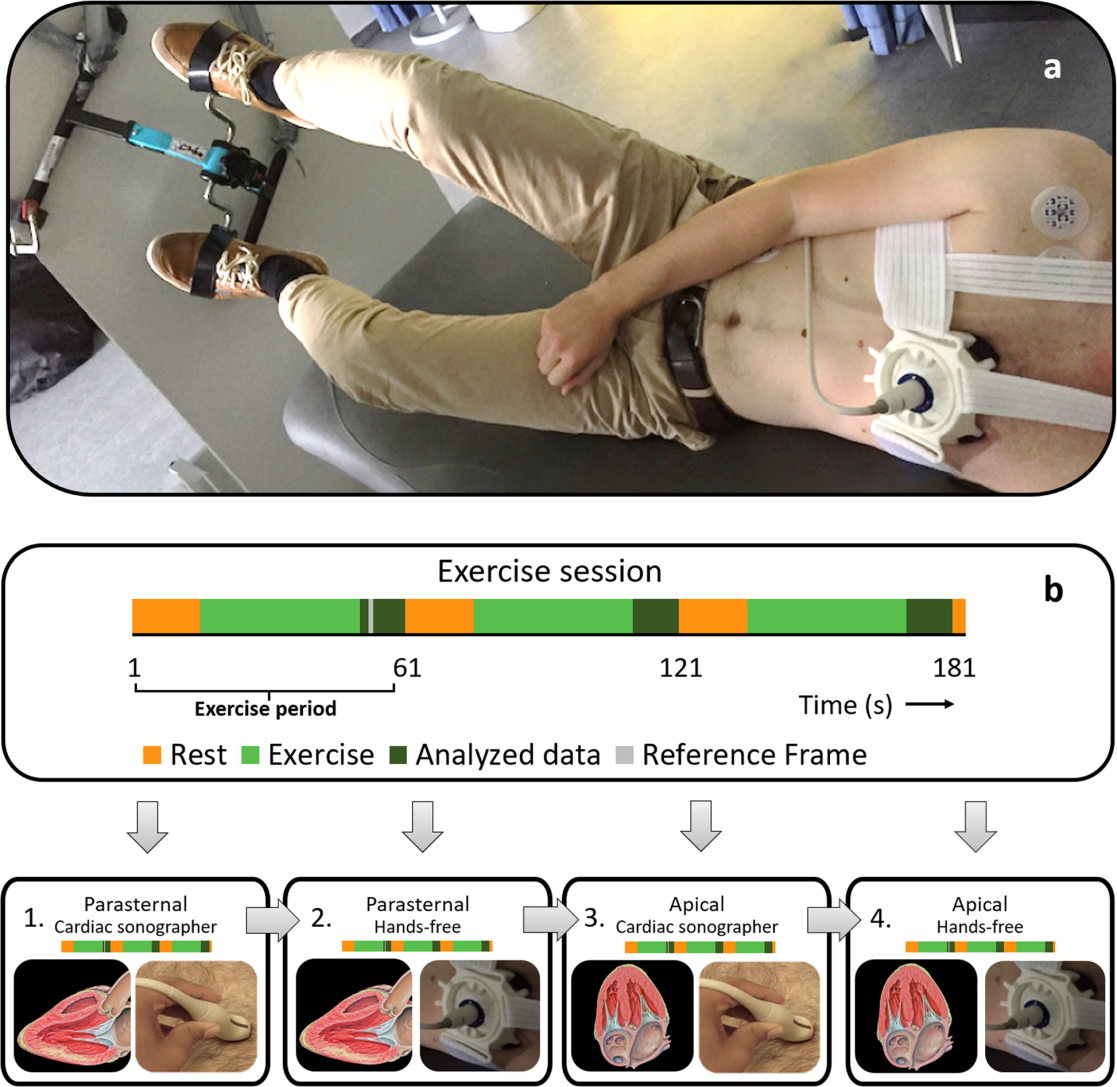
Experimental setup and acquisition protocol. **a** The semi-supine exercise setup in which the probe is fixated to acquire hands-free images of the heart. **b** The acquisition protocol. (1) Images were acquired manually in the parasternal view, followed by (2) hands-free measurements. Subsequently, (3) the manual and (4) hands-free acquisitions were obtained in the apical view [26–28]. The images of the last ten seconds of every exercise period were used for further assessment

Continuous echocardiography acquisitions were obtained during exercise. The subjects were tilted towards the left lateral decubitus position to improve the acoustic windows, induced by the pull of gravity on the heart. Additionally, this position provided a gravitational force, which would also be present in upright exercise on the probe and the fixation device, though in a different direction (Fig. 1a). Upper body motions, as observed in clinical stress tests, were mimicked using an exercise peddler with a digital display (drive, Germany) (Fig. 1a).

Ultrasound data were recorded using a phased array ultrasound transducer (PA230E, 1.0-4.0 MHz frequency range) connected to a Mylab70 scanner (ESAOTE Europe, Maastricht, the Netherlands). The images were collected in B-mode at a frame rate of 51-66 Hz whilst conserving all imaging settings during the evaluation of each participant per imaging window. Electrocardiograms were recorded by the scanner using a 4-lead ECG.

The ultrasound data were transferred to an external computer using the Digital Imaging and Communications in Medicine (DICOM) format to be analyzed in MATLAB (MATLAB 2016b, MathWorks, Natick, MA, USA). The files contained frame rates of 25 Hz with a resolution of 480x616 pixels with pixel dimensions ranging from 0.25 to 0.37 mm, depending on the imaging depth.

The exercise protocol for each separate continuous acquisition had a duration of three minutes, during which the subjects cycled at 80 RPM against a minor load for three periods of 45 seconds, with 15 seconds of rest prior to each exercise period (Fig. 1b). Each participant performed the exercise protocol four times. In the first exercise session, manual acquisitions were captured continuously in the parasternal long-axis view by a trained cardiac sonographer. In the second session, the transducer was fixated in an approximately identical position using a specially designed, CE-marked cardiac probe fixation device (ProbeFix, USONO, Eindhoven, The Netherlands). After initiation of the measuring period, no further positional adjustments of the transducers were allowed. Subsequently, the apical view four chamber view was observed during the third and fourth exercise sessions. Again, manual acquisitions were obtained first to assess the acoustic window and maximal attainable image quality, followed by hands-free measurements. In between the sessions, five minutes of rest were included for the subjects to recover completely.

### Data analysis algorithms

The algorithms used to analyze the acquisitions were implemented in MATLAB (MATLAB 2018b, MathWorks, Natick, MA, USA).

#### Image decomposition

**Fig. 2:**
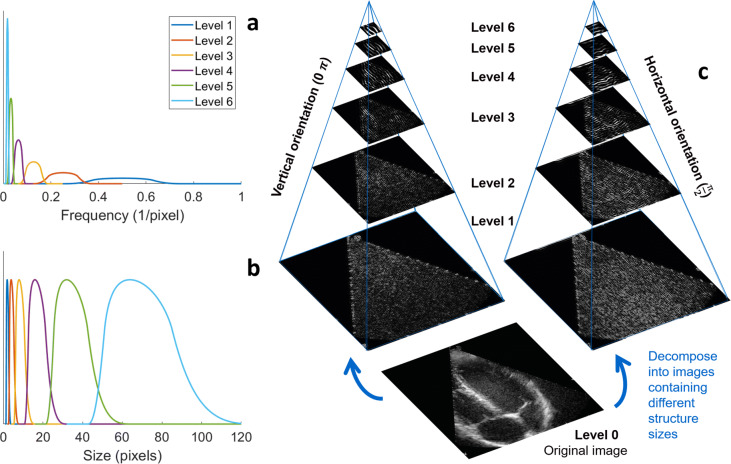
Steerable pyramid using complex, oriented wavelets. **a** The frequency subbands extracted of the six levels, and **b** the corresponding extracted image structural size ranges in pixels. The pixel dimensions ranged between 0.25 and 0.37 mm, depending on the image depth of the acquisition. **c** An example of the decomposition of an image into six levels, each containing a specific range of image structure sizes. For simplicity, only two of the sixteen orientations are illustrated (left = 0 *π* and right = $\frac {\pi }{2}$)

For the FOV stability analysis methods, and the elimination of frames showing A-lines generated by lung tissue, the ultrasound images were decomposed into a set of images, each containing image structures of a different size range (Fig. 2c). Accordingly, the influence of the image variance induced by noise and ultrasound speckle could be eliminated from the FOV stability analysis through nullification of the lower pyramid levels. In addition, detection of anatomical structures within a specific size range (and orientation) could be performed more easily (e.g. A-lines, heart valves, interventricular septum).

The decomposition was performed using a steerable pyramid which employed complex and oriented wavelets [29]. The wavelets resulted from the application of a low pass filter followed by high pass filtering. A different frequency subband was extracted by each wavelet from the frequency domain of the image (Fig. 2a), which corresponded to a structural size range in the spatial domain (Fig. 2b). Furthermore, oriented wavelets were utilized to simplify extraction and detection of specific image structures (i.e. the septum is orientated predominantly in a vertical direction, whereas A-lines are primarily horizontally oriented). In this method, complex wavelets were utilized instead of real wavelets, since most structural information is embedded in the relative phase patterns rather than the amplitude of the wavelet coefficients [30]. In this article, the frames were decomposed into six levels with 16 orientations, increasing with $\frac {\pi }{16}$.

#### Frame selection

**Fig. 3:**
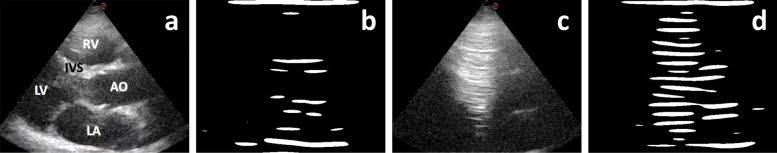
Detection of A-lines for frame exclusion. **a** A representative image of a parasternal long axis image (AO=aorta, L=left, R=right, A=atrium, V=ventricle and IVS=interventricular septum), and **b** the absence of A-lines in its reconstruction. **c** A frame in which the lung is blocking the ultrasound waves, and **d** portraying the detected A-lines

In the FOV stability analysis, a minimal influence of FOV variation induced by physiological changes was ensured by selecting frames obtained during steady-state and excluding frames depicting respiration- and cardiac contraction-induced motion as much as possible. Accordingly, only end-diastolic frames were included from the final 10 seconds of every exercise period, since during end-diastole the contraction velocity is minimal, whereas during end-systole the heart changes shape rapidly. Furthermore, ultrasound images displaying A-lines were automatically discarded. The presence of A-lines indicated concealment of the heart by lung tissue. A-lines are artifacts caused by vibration of the ultrasound waves between the proximal edge of air pockets in the lung and the ultrasound transducer. Hence, they are characterized by horizontal stripes appearing at regular intervals (Fig. 3c). For the automatic detection of A-lines, first, decomposition of the frames as described previously was performed (Fig. 2). In the dataset used the A-lines were visible in level four with orientation angle $\frac {\pi }{2}$. A frame containing A-lines was classified as such when its thresholded version, using Otsu’s thresholding method [31], exhibited at least four horizontal structures separated by equal interval distances in the upper half of the image (Fig. 3d).

#### FOV stability analysis method I: image structural similarity

The assessment of (regional) image similarity was performed using the complex wavelet structural similarity index method (CW-SSIM), which is a full-reference method. The CW-SSIM is based on human vision and compares the image structure defined as contrast (variance), luminosity (average pixel intensities), and image structures (covariance) to a chosen reference image [32].

In ultrasound data, the CW-SSIM has proven to be a suitable full-reference method [33], since it compares only the lowest frequencies for all orientations. Hence, the method a) excludes noise as much as possible, b) primarily compares the more dominant image structures and c) makes use of the fact that lower frequencies can be determined with a higher precision.

The CW-SSIM indices were computed for the selected frames with respect to the chosen reference frame. The reference frame was an end-diastolic frame with an adequate image quality captured during the final ten seconds of the first exercise period. Prior to the CW-SSIM analysis, the images were decomposed as previously described (Fig. 2). The CW-SSIM is defined as:

1 $$ S(c_{I},c_{II})\ = \frac{2 | \sum c_{I,i} \ c^{\ast}_{II,i}|}{\sum |c_{I,i} |^{2} + \sum |c_{II,i}|^{2} }   $$

with *c*_*I*_={*c*_*I*,*i*_|*i*=1,...,*N*} and *c*_*II*_={*c*_*II*,*i*_|*i*=1,...,*N*} the wavelet coefficients acquired from identical spatial positions in the lowest wavelet sub-band of the reference frame and the image it is being compared to, and *c*^∗^ the complex conjugate of *c* [32]. Subsequently, the CW-SSIM index for each oriented subband from level six is calculated by combining the localized indexes into a scalar utilizing a 7 x 7 Gaussian filter with the standard deviation equaling a quarter of the size of the evaluated sub-band [32]. Finally, the mean of these local indices is taken, resulting in the CW-SSIM index, which describes the similarity of the two images. In case the two images are identical, a CW-SSIM index of 1 will be computed. However, when the image structures differ completely, the CW-SSIM index will drop to zero. Furthermore, the CW-SSIM index is insensitive to small rigid translations and pixel intensity changes, and an intensity change of 10% only results in a CW-SSIM index decrease from 1 to a value equal to or higher than 0.996 [32].

A representative CW-SSIM index for each exercise period was obtained by computing the median of the selected frames. Furthermore, the variability of each CW-SSIM dataset was determined by taking the median of the interquartile ranges of all exercise periods. The median and interquartile range represent the most prevalent data, whereas the mean and standard deviation penalize outliers. Hence, in this study the median and interquartile range were chosen to represent the CW-SSIM values, since image structural similarity is strongly affected by partial dislocation of the transducer onto a rib, which occurred only sporadically during a deep in- or exhalation in most measurements.

#### FOV stability analysis method II: cardiac feature consistency

As a second FOV stability quantification method, temporal deviations of the cardiac structures were evaluated. An automatic evaluation of the orientation and curvature of the interventricular septum was performed in the apical four chamber view. First, the selected frames were decomposed into a set of subband images containing different sizes of images structures as previously explained (Fig. 2). The subbands encompassing the frequencies making-up the cardiac walls and valves were selected and merged together using the inverse fast wavelet transform [29, 34]. This resulting image predominantly displays the cardiac structures of interest, whilst suppressing ultrasound speckle and noise. Subsequently, pixels representing the septum were selected by automatically locating the valves and vertical walls, by searching for the crossing of the valves and the septal wall. The section of the septum proximal to the transducer was neglected, since this portion was not consistently visible in the frames. Through the pixel collection representing the interventricular septum, a straight line and a parabola was fit to compute the tilt and curvature of the septum, respectively. The tilt was defined as the slope of the fitted line with respect to a vertical line, and the curvature was obtained using the following equation:

2 $$ c=\frac{y^{\prime\prime}}{(1+y'^{2})^{\frac{3}{2}}}  $$

with *c* the curvature, and *y* the equation representing the parabola fitted through the septum. The origin of the coordinate system was defined as the maximum of the parabola. The derivatives of *y* were computed with respect to the tangent of the maximum of the parabola. The absolute alteration in septum curvature and tilt were determined by subtracting the average of the first exercise period from the average of the last exercise period.

#### Statistical evaluation

Significant differences between the images acquired by the fixated transducer and the trained sonographer were calculated using the two-sided paired t-test to neglect body-shape, upper body motion speed and amplitude, respiration-induced motion, and other non-fixation-related factors that might influence the measurement results. An *α* of 0.05 was used. Normal distribution for the paired t-test was tested using the Shapiro-Wilk test. Groups not displaying a normal distribution were statistically evaluated using the two-sided Wilcoxon Signed Rank-Sum test. The two-sided power was assessed for the normally distributed data sets with no significant difference to determine the percentage of chance to reject the null hypothesis in case the null hypothesis was false. A power of 0.80 was regarded sufficient.

#### Hands-free septal longitudinal strain

Septal longitudinal strain was computed at the end of each exercise period to determine the feasibility and consistency of hands-free functional measurements. The analysis was performed using speckle-tracking software, which is an image analysis method able to estimate the deformation of tissue by calculating the most likely displacement of pixel groups between frames [35, 36]. Three individual heart cycles in the apical view were analyzed at the end of each exercise period, starting at end-diastole. Respiration-induced motion of the heart and the limited FOV in both the images acquired by the sonographer and hands-free meant only the interventricular septum was visible continuously in all acquisitions. Accordingly, the septal wall was manually delineated in the end-diastolic frames. Subsequently, a mesh of 11 by 31 points was generated covering the segmented area. Inter-frame pixel group displacements were calculated in the axial and lateral directions for each mesh point sequentially, using a coarse-to-fine block-matching algorithm, which employs the cross-correlation function [35]. Cumulative longitudinal strain was calculated based on the deformation of the mesh with respect to the end-diastolic frame.

## Results

The trained sonographer was able to acquire ten and eleven adequate measurements in the apical and parasternal view in the twelve subjects, respectively. In the remainder of the cases the acoustic windows were inadequate for left ventricle visualization. In both views, nine adequate measurements were able to be obtained using the probe fixation device. The views unable to be assessed hands-free were caused by dislocation of the transducer behind a rib. Consequently, only the data sets that contained both hands-free and manually acquired images were used for further analysis.

***FOV stability analysis method I: image structural similarity***

**Fig. 4:**
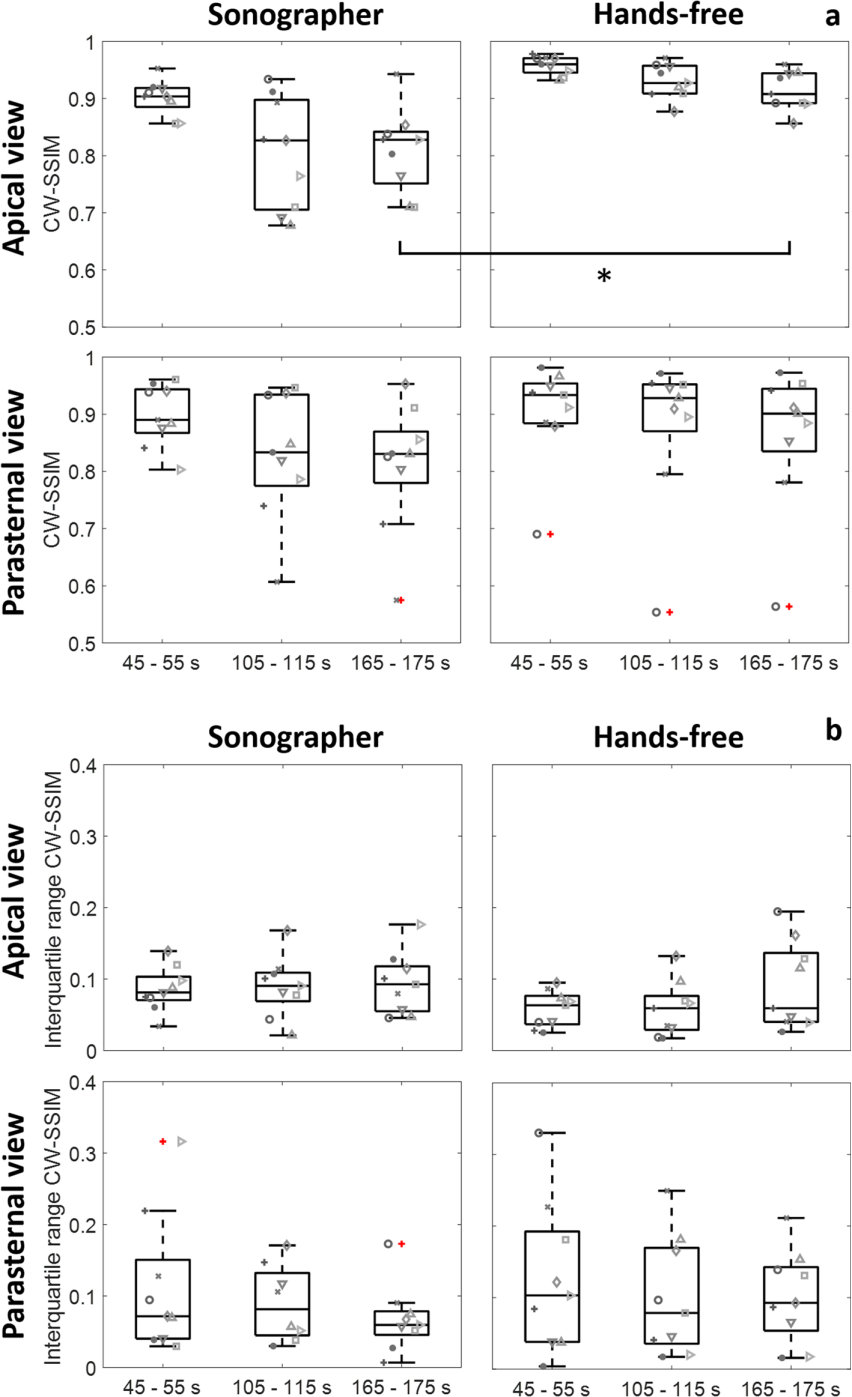
CW-SSIM indices and interquartile ranges of the apical and parasternal windows. **a** The CW-SSIM indices of the hands-free and manually obtained datasets. In the apical four chamber view, a significant difference was detected between the median CW-SSIM indices of the third exercise periods. **b** The variability of the CW-SSIM data expressed using the interquartile ranges. In both views, the interquartile ranges appeared to be similar when comparing the hands-free and manually obtained acquisitions

The median CW-SSIM indices of the final ten seconds of the exercise periods are depicted in Fig. 4a. All datasets displayed a decrease in CW-SSIM index over time, though the hands-free datasets appeared to be more constant during the course of the measurement.

In the apical view, the CW-SSIM indices of the hands-free acquired data of the last exercise period were significantly higher compared to those obtained by the sonographer (*p*=0.0046), with the median CW-SSIM indices being 0.91 and 0.82, respectively.

In the parasternal view, in seven out of nine, the hands-free median CW-SSIM indices were higher in comparison to the manual acquisitions during the last exercise period. The hands-free outlier dataset was caused by respiration-induced out-of-plane motion of the heart, since the uncommonly short A-lines in these frames were undetected by the algorithm. The average CW-SSIM indices of the last exercise periods were 0.90 and 0.83 for the hands-free and manually obtained data, respectively. No significant difference was observed between the last exercise periods, though the computed power of 0.15 indicated a low probability to reject the null hypothesis in case the null hypothesis was false, in which the null hypothesis assumed the hands-free and manual data to be similar.

The interquartile ranges of the CW-SSIM dataset appeared to be similar when comparing the sonographer to the hands-free acquisitions (Fig 4b). In the apical view, the interquartile ranges of the hands-free and manual datasets were 0.06 and 0.09 on average, respectively. In the parasternal view, the interquartile ranges of the hands-free and manual data were 0.09 versus 0.07 on average, respectively. The interquartile ranges of the apical view CW-SSIM datasets were not normally distributed. Hence, the Wilcoxon signed rank-sum test was applied, whereas for the parasternal view the paired t-test was performed. No significant differences were found. The power of the parasternal view datasets was only 0.08. The power for the apical view could not be computed, since the data was not normally distributed.

***FOV stability analysis method II: cardiac feature consistency***

**Fig. 5:**
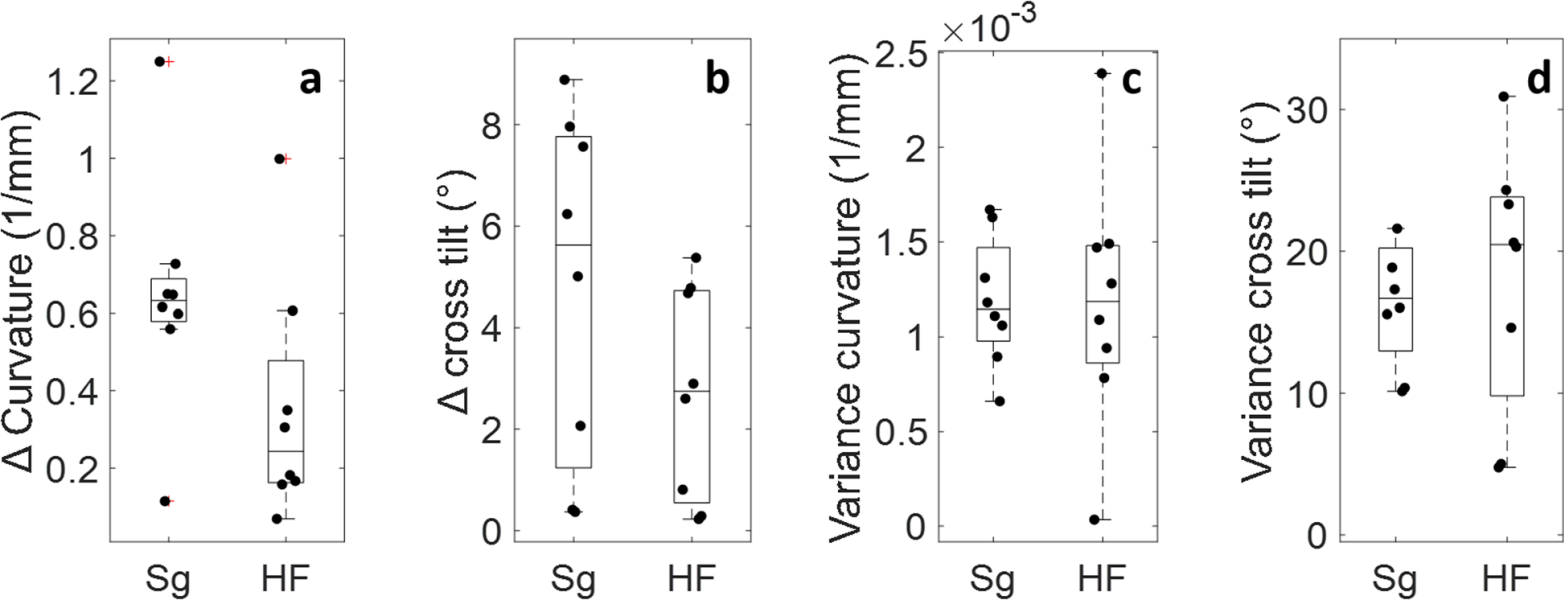
Alterations in septum curvature and tilt. **a** the absolute total deviation of the septum and **b** the tilt, and the variance in **c** septum curvature and **d** tilt. No significant differences were found, though the absolute alterations depicted in (**a**) and (**b**) appear to be lower in the hands-free data. The variances of the hands-free and manually acquired data (**c** and **d**) are similar

Alterations in the apical view four-chamber view were also quantified by evaluation of the septum curvature and tilt (Fig. 5). In the hands-free data, the absolute differences in septum curvature and tilt appeared to be smaller in comparison to the measurements taken by the sonographer (Figs. 5a and b). The variances in septum curvature were similar for the hands-free and manual data (Fig. 5c). In case of the septum tilt, on average, the cardiac sonographer appeared to have acquired images with less variation in interventricular septum tilt (Fig. 5d).

No significant differences were found between the hands-free acquisitions and the images obtained by the cardiac sonographer for any of the parameters, though the powers of these statistical computations were very low (Table 1).

**Table 1:** Powers of the data groups shown in the Figs. 4 and 5

	Power
CW-SSIM index Parasternal	0.15
Interquartile range CW-SSIM Parasternal	0.08
|*Δ* Curvature |	0.43
Curvature variance	0.16
|*Δ* Cross tilt |	0.26
Cross tilt variance	0.07

***Hands-free septal longitudinal strain***

**Fig. 6:**
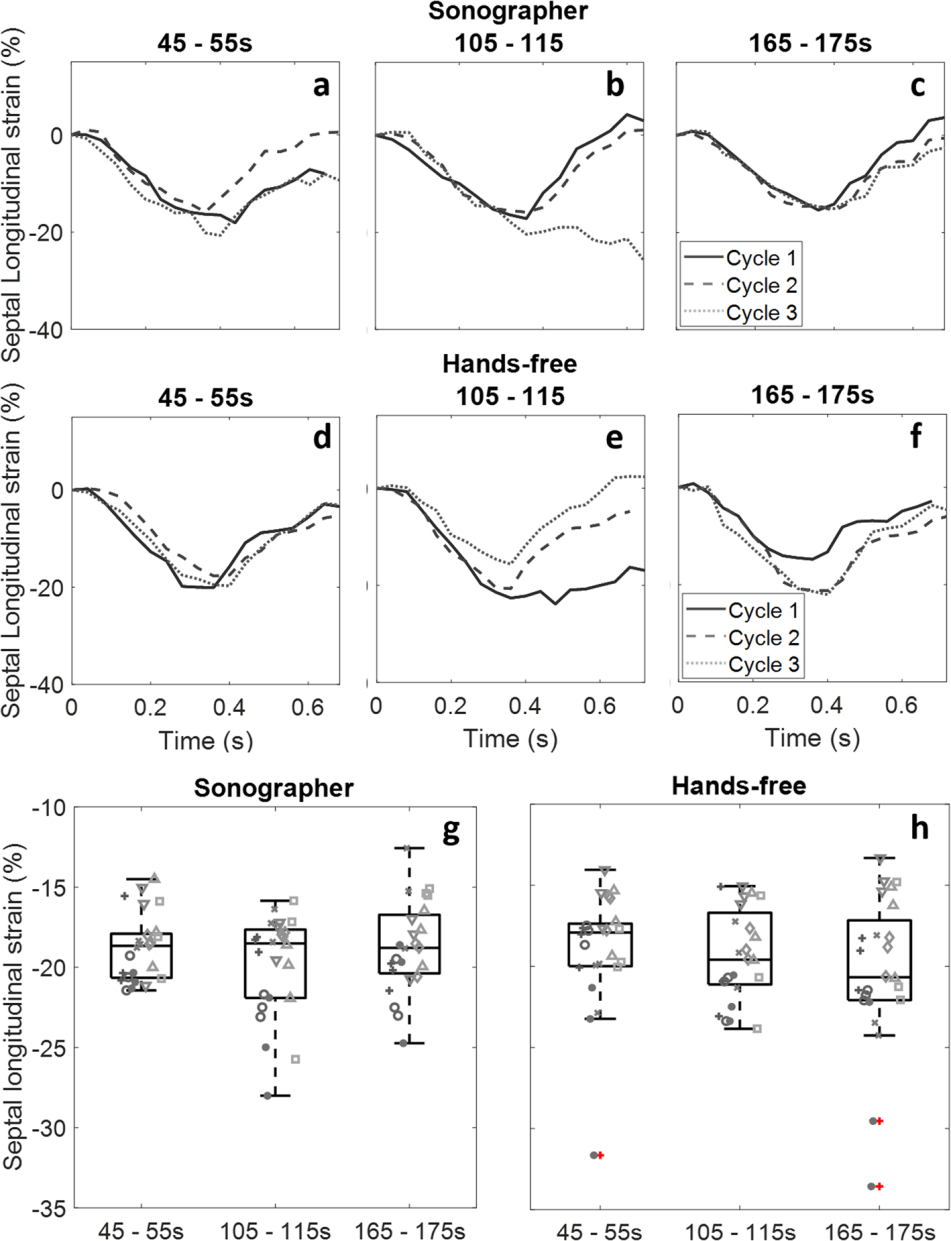
Septal longitudinal strain. **a**-**f** show representative longitudinal strain (%) curves acquired in the apical view from a single volunteer. **g**-**h** display the variability of septal longitudinal strain estimates acquired by the trained observer and of the hands-free acquisitions. Interquartile ranges are similar between both methods, although there is some variation in median values

The feasibility of functional ultrasound evaluation of the heart with a fixated transducer was demonstrated during cycling exercise echocardiography in nine volunteers. The computed longitudinal strains for each tracking point were averaged to give a single estimate of septal longitudinal strain per frame. The curves show good agreement between the sonographer and hands-free methods (representative curves are shown in Fig. 6a-f). Drift occurred during heart cycles where the heart moved out of view or out of plane, or when the lung moved into view. Examples of this drift can be seen in Fig. 6b and e. Peak septal longitudinal shortening during each cycle are illustrated in Fig. 6g and h. The median and interquartile ranges are similar for both methods, although a small increase in strain magnitude is seen for the hands-free acquisitions.

## Discussion

Field-of-view stability is a requirement for cardiac functional evaluation for both qualitative and quantitative measurements. During exercise stress echocardiography, FOV preservation and reproducibility is especially complicated and strenuous for the sonographer. Therefore, in this study, a demonstration of the FOV stability of hands-free echocardiography is presented. The hands-free acquisitions, obtained using a fixated transducer, were objectively compared to acquisitions acquired by a clinically trained sonographer in healthy volunteers during exercise stress echocardiography. Quantification of the FOV stability was computed using the CW-SSIM index, which is a suitable method for image structure comparison in ultrasound data [33]. Furthermore, the variability of the CW-SSIM indices and interventricular septal image features during the measurements was compared. Secondly, the total change in interventricular septal image features was assessed, being the curvature and orientation of the interventricular septum. Thirdly, the feasibility and consistency in longitudinal, interventricular septal strain was assessed.

Based on the CW-SSIM indices, the FOV of the hands-free acquisitions was found to be more stable than, or similar to, the manual data. The CW-SSIM values of the hands-free measurements after three periods of 45 seconds of exercise were significantly higher compared to the manual data in the apical view. In the parasternal images, the CW-SSIM indices of the hands-free data were higher on average, though no significant differences were observed. However, the power was lower than 0.80, implying the analysis of a bigger group of participants might have still revealed a significant difference.

Furthermore, the higher temporal consistency in the CW-SSIM indices of the hands-free data in both views might suggest the reproducibility of the hands-free data to be better in comparison to the manual acquisitions, though more extensive evaluation is necessary, which is outside the scope of the article. In the apical view, besides an improved FOV stability, the variability within the measurements seemed to be slightly lower or comparable. The interquartile ranges of the parasternal hands-free and manual data were also similar, though the variability of the hands-free data seemed to be slightly higher, possibly as a result of respiration-induced motion. In this study, the parasternal images were affected to a greater extent by respiration. In contrast to the fixated transducer, the sonographer may have been able to correct for the periodically respiration-induced motion of the chest and heart. However, over time the fixated transducer was able to preserve the imaging plane to a better extent, whereas the sonographer changed the FOV slightly and more abruptly. These respiration-induced out-of-plane motions might be counteracted by acquiring 3-D ultrasound images. In approximately one in five cases, the hands-free acquisitions were insufficient for further analysis, which is in accordance with literature about hands-free measurements [23, 24].

The CW-SSIM indices provide little information on the clinical relevancy of the hands-free data in comparison to the manual acquisitions. The FOV consistency might be high in the hands-free data, though a small transducer displacement might produce a clinically inadequate view. The parasternal view is predominantly used for valvular inspection, since the apex is the region containing most wall motion anomalies [8], which is not visible in the parasternal view. Accordingly, in this case, minor alterations are tolerated in the imaging plane of the parasternal view. In contrast, in the apical view four chamber view, inadequate imaging planes will negatively affect the computation of the functional parameters [10]. In addition, in stress echocardiography, changes in these functional parameters as a result of increased cardiac stress, are indicators for coronary disease. Hence, the imaging plane should be consistent over the entire measuring period for accurate functional parameter investigation. Therefore, an assessment of the interventricular septum curvature and tilt consistency was performed. No significant differences in interventricular septum tilt and curvature between the hands-free and manual datasets were observed. However, the data indicate a lower total septum curvature and tilt alteration over the entire measurement period. In addition, the variance of the curvature and tilt seemed to be similar when comparing the acquisition methods. Therefore, in the apical view, the clinical value of the hands-free data can be inferred to be higher compared to the acquisitions of the trained cardiac sonographer, though more measurement should be performed for conformation, as the statistical power of these investigations was low.

The feasibility of hands-free functional evaluation of the left ventricle during exercise stress echocardiography was assessed through computation of longitudinal strain of the septal wall, since septal strain measurements have shown to be the best clinical marker for left ventricle functionality [19]. We hypothesized that deviation (or a lack thereof) in strain magnitude between different heart cycles and exercise periods would provide information on the FOV stability. In the case of a consistent FOV, strain parameters were expected to remain roughly constant. Inconsistent FOV leads to drift in strain estimates due to obstruction of the region of interest, and differences in strain magnitude due to out of imaging plane motion of the myocardium. Good agreement was found between the sonographer and hands-free acquisitions for both the strain curves and peak septal longitudinal strain, with hands-free strain at least as consistent as the sonographer.

The biggest limitation of the study was the relatively small number of subjects. In the future, a larger study should be performed for the comparison between manual and hands-free acquisitions in stress echocardiography. Nevertheless, this initial study demonstrates that the FOV in hands-free exercise stress echocardiography is at least as stable as manual acquisitions.

The increased or equivalent FOV stability observed in the hands-free measurements in this study is encouraging for the use of hands-free devices in upright stress echocardiography in future studies and in the clinic. In upright exercise, higher heart rates and workload can be achieved, which increases the sensitivity of the test [10–12]. Additionally, it better represents daily activities. However, hands-free, upright cardiac acquisitions could be limited by the reduction in acoustic window size and the less optimal heart position compared to a supine-like position. Fortunately, Salden et al. [23] has demonstrated the feasibility of hands-free acquisitions in upright exercise. However, in this research, supine exercise was undertaken, since a comparison to clinically comparable acquisitions was made.

Additionally, enhanced reproducibility may provide another benefit; high inter-observer variability is a major problem in ultrasound assessments, which is a result of ineffective training in image interpretation, and observer subjectivity and bias [10, 37, 38]. In case interval acquisitions are obtained, acquiring identical imaging planes is a difficult task. Hence, the FOV stability of interval measurements is expected to be lower than in the continuously obtained manual acquisitions in this study. Transducer fixation can overcome these issues, since the imaging plane will be preserved during the measurement period. However, the feasibility of hands-free imaging in women should still be investigated. The chest sizes and shapes of women deviate to a greater extent in women than in men, and their breasts might influence the FOV consistency, especially during exercise. In the newer stress echocardiography protocols, lung water detection using B-lines, left ventricle contractile reserve, and Doppler-based coronary micro-circulation evaluation (i.e. coronary flow velocity reserve) are added to the mitral valve and wall motion abnormalities assessment [7]. These extra parameters provide a better evaluation of vulnerability to adverse events, and it is a more omnivorous approach resulting in a more complete diagnostic overview of heart failure or coronary artery disease patients [1–6]. B-line observation can be easily performed in the hands-free stress echocardiography set-up, since the probe can be dismounted and reattached quickly. The images required for left ventricle contractile reserve assessment are already obtained during the regular stress exam. Hands-free computation of the coronary flow velocity reserve will be most challenging, since these acquisitions are already technically challenging and require state-of-the art ultrasound equipment [4, 5]. Additionally, the images are obtained in the low parasternal long-axis view and/or modified two-, three-, or four-chamber apical view. Hence, the position of the probe and its fixation device would need to be modified, which further complicates these measurements. Therefore, if the coronary flow velocity reserve is only measured prior to exercise and near peak exercise, then hands-free acquisitions should be feasible. However, this feasibility should be analyzed in future studies. Besides continuous intensive care acquisitions [24] and exercise stress echocardiography, continuous intra- and postoperative transthoracic sonography could be beneficial, since it facilitates the detection of ischemia cases that are challenging to diagnose using an ECG or other standard hemodynamic monitored parameters [39]. Furthermore, the alternative, transesophageal echocardiography, is accompanied with risks and is no longer possible after intubation [18, 40]. The application of transthoracic echography has already been proven to be successful in directly preventing perioperative adverse events and has been proven feasible in more than 90% of the evaluated operations [17, 18]. However, these measurements were performed on an interval basis. Hence, the feasibility of continuous acquisitions is unclear.

## Conclusions

Hands-free acquisitions provide at least equally good field-of-view stability, and improved or comparable image consistency over time. The variability within measurements was similar. Furthermore, the acquisition of hands-free strain data has been shown to be feasible. Additionally, the hands-free strain was at least as consistent as the measurements performed by the sonographer. Consequently, an improved sensitivity of cardiac stress tests might be obtained for wall motion assessment and valve functionality evaluation. Hands-free echocardiography might provide a higher reproducibility with additional applications possible in intraoperative and intensive care for functional cardiac evaluation.

## Data Availability

The datasets used and/or analyzed during the current study are available from the corresponding author on reasonable request.

## Abbreviations

CW-SSIM: Complex wavelet structural similarity index method
FOV: Field-of-view
DICOM: Digital imaging and communications in medicine

## References

[1] Scali MC, Zagatina A, Ciampi Q, Cortigiani L, D’Andrea A, Djordjevic-Dikic A, Merlo PM, Lattanzi F, Simova I, Monte I (2019). The functional meaning of b-profile during stress lung ultrasound. JACC Cardiovasc Imaging.

[2] Scali MC, Cortigiani L, Simionuc A, Gregori D, Marzilli M, Picano E (2017). Exercise-induced b-lines identify worse functional and prognostic stage in heart failure patients with depressed left ventricular ejection fraction. Eur J Heart Fail.

[3] Cortigiani L, Huqi A, Ciampi Q, Bombardini T, Bovenzi F, Picano E (2018). Integration of wall motion, coronary flow velocity, and left ventricular contractile reserve in a single test: prognostic value of vasodilator stress echocardiography in patients with diabetes. J Am Soc Echocardiogr.

[4] Zagatina A, Zhuravskaya N (2017). The additive prognostic value of coronary flow velocity reserve during exercise echocardiography. Eur Heart J Cardiovasc Imaging.

[5] Ciampi Q, Zagatina A, Cortigiani L, Gaibazzi N, Daros CB, Zhuravskaya N, Wierzbowska-Drabik K, Kasprzak JD, e Silva JLdC, D’Andrea A (2019). Functional, anatomical, and prognostic correlates of coronary flow velocity reserve during stress echocardiography. J Am Coll Cardiol.

[6] Lowenstein J, Tiano C, Marquez G, Presti C, Quiroz C (2003). Simultaneous analysis of wall motion and coronary flow reserve of the left anterior descending coronary artery by transthoracic doppler echocardiography during dipyridamole stress echocardiography. J Am Soc Echocardiogr.

[7] Picano E, Ciampi Q, Wierzbowska-Drabik K, Urluescu M-L, Morrone D, Carpeggiani C (2018). The new clinical standard of integrated quadruple stress echocardiography with abcd protocol. Cardiovasc Ultrasound.

[8] Marwick TH (2003). Stress Echocardiography: Its Role in the Diagnosis and Evaluation of Coronary Artery Disease.

[9] Attenhofer CH, Pellikka PA, Oh JK, Roger VL, Sohn D-W, Seward JB (1996). Comparison of ischemic response during exercise and dobutamine echocardiography in patients with left main coronary artery disease. J Am Coll Cardiol.

[10] Pellikka PA, Nagueh SF, Elhendy AA, Kuehl CA, Sawada SG (2007). American society of echocardiography recommendations for performance, interpretation, and application of stress echocardiography. J Am Soc Echocardiogr.

[11] MacWilliam J (1933). Postural effects on heart-rate and blood-pressure. Quarterly Journal of Experimental Physiology: Translation and Integration.

[12] Proctor DN, Sinning WE, Bredle DL, Joyner MJ (1996). Cardiovascular and peak vo2 responses to supine exercise: effects of age and training status. Med Sci Sports Exerc.

[13] Evans K, Roll S, Baker J (2009). Work-related musculoskeletal disorders (wrmsd) among registered diagnostic medical sonographers and vascular technologists: a representative sample. J Diagn Med Sonography.

[14] Simonsen JG, Axmon A, Nordander C, Arvidsson I (2017). Neck and upper extremity pain in sonographers–associations with occupational factors. Appl Ergon.

[15] Coffin CT (2014). Work-related musculoskeletal disorders in sonographers: a review of causes and types of injury and best practices for reducing injury risk. Rep Med Imaging.

[16] Modesto KM, Rainbird A, Klarich KW, Mahoney DW, Chandrasekaran K, Pellikka PA (2003). Comparison of supine bicycle exercise and treadmill exercise doppler echocardiography in evaluation of patients with coronary artery disease. Am J Cardiol.

[17] Ashley E, Niebauer J (2004). Chapter 4: Understanding the echocardiogram: In cardiology explained.

[18] Jørgensen MRS, Juhl-Olsen P, Frederiksen CA, Sloth E (2016). Transthoracic echocardiography in the perioperative setting. Curr Opin Anesthesiol.

[19] Dandel M, Lehmkuhl H, Knosalla C, Suramelashvili N, Hetzer R (2009). Strain and strain rate imaging by echocardiography-basic concepts and clinical applicability. Curr Cardiol Rev.

[20] Hori K, Matsuura T, Mori T, Nishikawa K (2015). Usefulness and growing need for intraoperative transthoracic echocardiography: a case series. BMC Anesthesiol.

[21] Nakashiki K, Kisanuki A, Otsuji Y, Yoshifuku S, Yuasa T, Takasaki K, Kuwahara E, Yu B, Uemura T, Mizukami N (2006). Usefulness of a novel ultrasound transducer for continuous monitoring treadmill exercise echocardiography to assess coronary artery disease. Circ J.

[22] Chandraratna P, Gajanayaka R, Makkena SM, Wijegunaratne K, Hafeez H, Vijayasekaran S, Ali A. (2010). “hands-free” continuous echocardiography during treadmill exercise using a novel ultrasound transducer. Echocardiography.

[23] Salden O, Van Everdingen W, Spee R, Doevendans P, Cramer M (2018). How i do it: feasibility of a new ultrasound probe fixator to facilitate high quality stress echocardiography. Cardiovasc Ultrasound.

[24] Blans M, Bosch F, van der Hoeven J (2019). The use of an external ultrasound fixator (probefix) on intensive care patients: a feasibility study. Ultrasound J.

[25] Bouwmeester S, de Kleijn M, van Wijngaarden J, Houthuizen P (2019). The use of a probe stabilizer to reduce musculoskeletal overload of ultrasound operators in routine diagnostic echocardiographic imaging. J Ultrason.

[26] Qasim A. Transducer position of the parasternal long axis [Digital image]. 2018. https://echocardiographer.org/TTE.html.

[27] Lynch PJ, Jaffe CC. Apical four-chamber echocardiographic view of the normal heart [Digital image]. 2006. https://commons.wikimedia.org/wiki/File:Heart_apical_4c_anatomy.jpg.

[28] Lynch PJ, Jaffe CC. Heart normal left parasternal long axis echocardiography view [Digital image]. 2006. https://commons.wikimedia.org/wiki/File:Heart_left_parasternal_long_axis.jpg.

[29] Portilla J, Simoncelli EP (2000). A parametric texture model based on joint statistics of complex wavelet coefficients. Int J Comput Vis.

[30] Oppenheim AV, Lim JS (1981). The importance of phase in signals. Proc IEEE.

[31] Otsu N (1979). A threshold selection method from gray-level histograms. IEEE Trans Syst Man Cybern.

[32] Sampat MP, Wang Z, Gupta S, Bovik AC, Markey MK (2009). Complex wavelet structural similarity: A new image similarity index. IEEE Trans Image Process.

[33] Xu K, Liu X, Cai H, Gao Z. Full-reference image quality assessment-based b-mode ultrasound image similarity measure. arXiv preprint arXiv:1701.02797. 2017.

[34] Simoncelli EP, Freeman WT (1995). The steerable pyramid: A flexible architecture for multi-scale derivative computation. Proceedings., International Conference on Image Processing.

[35] Lopata RG, Nillesen MM, Hansen HH, Gerrits IH, Thijssen JM, De Korte CL (2009). Performance evaluation of methods for two-dimensional displacement and strain estimation using ultrasound radio frequency data. Ultrasound Med Biol.

[36] Fixsen LS, de Lepper AG, Strik M, van Middendorp LB, Prinzen FW, van de Vosse FN, Houthuizen P, Lopata RG (2019). Echocardiographic assessment of left bundle branch–related strain dyssynchrony: a comparison with tagged mri. Ultrasound Med Biol.

[37] Jacobson JA (2009). Musculoskeletal ultrasound: focused impact on mri. Am J Roentgenol.

[38] Giannakou E, Aggeloussis N, Arampatzis A (2011). Reproducibility of gastrocnemius medialis muscle architecture during treadmill running. J Electromyogr Kinesiol.

[39] Kumar A, Anel R, Bunnell E, Habet K, Zanotti S, Marshall S, Neumann A, Ali A, Cheang M, Kavinsky C (2004). Pulmonary artery occlusion pressure and central venous pressure fail to predict ventricular filling volume, cardiac performance, or the response to volume infusion in normal subjects. Crit Care Med.

[40] Jensen M, Sloth E, Larsen KM, Schmidt MB (2004). Transthoracic echocardiography for cardiopulmonary monitoring in intensive care. Eur J Anaesthesiol.

